# How can health economics be used in the design and analysis of adaptive clinical trials? A qualitative analysis

**DOI:** 10.1186/s13063-020-4137-2

**Published:** 2020-03-06

**Authors:** Laura Flight, Steven Julious, Alan Brennan, Susan Todd, Daniel Hind

**Affiliations:** 1 School of Health And Related Research, University of Sheffield, Sheffield, UK; 2 Department of Mathematics and Statistics, University of Reading, Reading, UK

**Keywords:** Adaptive design, Health economics, Value of information, Cost-effectiveness, Recommendations

## Abstract

**Introduction:**

Adaptive designs offer a flexible approach, allowing changes to a trial based on examinations of the data as it progresses. Adaptive clinical trials are becoming a popular choice, as the prudent use of finite research budgets and accurate decision-making are priorities for healthcare providers around the world. The methods of health economics, which aim to maximise the health gained for money spent, could be incorporated into the design and analysis of adaptive clinical trials to make them more efficient. We aimed to understand the perspectives of stakeholders in health technology assessments to inform recommendations for the use of health economics in adaptive clinical trials.

**Methods:**

A qualitative study explored the attitudes of key stakeholders—including researchers, decision-makers and members of the public—towards the use of health economics in the design and analysis of adaptive clinical trials. Data were collected using interviews and focus groups (29 participants). A framework analysis was used to identify themes in the transcripts.

**Results:**

It was considered that answering the clinical research question should be the priority in a clinical trial, notwithstanding the importance of cost-effectiveness for decision-making. Concerns raised by participants included handling the volatile nature of cost data at interim analyses; implementing this approach in global trials; resourcing adaptive trials which are designed and adapted based on health economic outcomes; and training stakeholders in these methods so that they can be implemented and appropriately interpreted.

**Conclusion:**

The use of health economics in the design and analysis of adaptive clinical trials has the potential to increase the efficiency of health technology assessments worldwide. Recommendations are made concerning the development of methods allowing the use of health economics in adaptive clinical trials, and suggestions are given to facilitate their implementation in practice.

## Introduction

Decision-makers around the world are faced with limited budgets for funding health research, such as new clinical trials, and funding health technologies, such as new drugs [1–3]. Resources spent on funding new research may otherwise be spent directly on patient care, and so it is desirable to consider the cost-effective use of limited resources. The methods of health economics facilitate resource allocation decisions by evaluating the maximum health gained for the resources spent [4].

Cost-effectiveness plays an important role at two points in the health technology assessment (HTA) process: to determine whether it is cost-effective to fund a new health technology and whether it is cost-effective to fund a piece of research. For the first of these questions, cost-effectiveness analysis compares the costs and benefits (often measured using quality-adjusted life years (QALYs)) of competing health technologies to identify which represent value for money [4]. For the second type of evaluation, value of information analysis (VOIA) assesses whether it is worthwhile collecting further information, such as performing further clinical trials, to reduce decision uncertainty [5, 6]. Amongst other inputs, a reliable health economic analysis requires an accurate estimate of the treatment effect and associated confidence interval, often provided by a clinical trial.

Adaptive designs are one innovative approach to conducting a clinical trial. Unlike a traditional fixed sample size design, data are examined as the trial progresses to inform modifications to the trial. This can potentially save time and resources, as well as prevent patients from being needlessly randomised [7–9]. The number of trials using adaptive methods has increased from 11 per 10,000 registered trials between 2001 and 2005 to 38 per 10,000 registered trials between 2012 and 2013 [10].

Adaptive designs and their implementation are commonly based on demonstrating clinical effectiveness. Despite its importance, cost-effectiveness is often a secondary consideration [11]. It is currently unclear what impact the use of an adaptive design has on a health economic analysis. Additionally, opportunities are potentially being missed to incorporate health economics into the design and analysis of adaptive clinical trials. The objective of this paper is to report the views of stakeholders on the use of health economics in adaptive clinical trials.

## Methods

We adopted a pragmatic approach to understanding issues around the use of health economics in adaptive designs [12]. An initial thematic framework was developed, based on the research aims, literature and discussions with our public advisory group (a group of members of the public advising on the research). Initial themes identified included the following: Pre-specification of Health Economic Analysis; Private Sector Attitude; Aims of a Clinical Trial and Advantages and Limitations of Adaptive Designs. Once data collection was under way, we amended the framework based on inferences about participant perspectives with no obvious place in the initial framework. The final framework in Table 1 condensed the themes into three main headings reflecting the key areas of interest: ethical, methodological and practical issues.

**Table 1:** Final thematic framework applied to the data collected from interview and focus groups HTA; health technology assessment

**Theme**	**Subtheme**	**Description**	**Example**
Methodological	Development	Important statistical issues to consider when developing the use of adaptive designs and health economics in HTA	How to handle bias in the analysis of group sequential trials Advantages and disadvantages of using adaptive designs and health economics together in clinical trials Issues with cost data that need to be considered when developing the methods
	Current use	How are adaptive designs, economic evaluations and value of information analysis methods currently used in practice and what are their advantages and disadvantages?	Use of value of information analysis methods is limited in practice because it is often perceived as complicated
	Experience and knowledge	What experience and knowledge do the participants have of these methods and where has this come from?	Members of the public may rely on the media for information about healthcare decision-making
Ethical	Planning	Ethical issues that should be considered when planning trials (before they begin) using adaptive designs and health economics	The motivations of patients for being involved in trials can differ, and so using cost-effectiveness as part of a trial may or may not influence whether people want to take part The aims of a clinical trial from all stakeholder perspectives (when talking at a societal/personal level rather than statistical) The context where these methods are appropriate from an ethical point of view, e.g. end of life Historical case studies have influenced changes in policy and how we run clinical trials, and methods need to reflect this
	Implementation and conduct	Ethical issues that should be considered when conducting or implementing trials using adaptive designs and health economics in the real world (during the trial)	The methods could allow the assumption of equipoise to be compromised at interim analyses Protecting participants in trials if the trial stops How the DMEC would be composed in these trials
	Documenting and reporting	Ethical issues that should be considered when writing documentation for trials using adaptive designs and health economics (not just reporting after the trial has ended)	Informed consent is key to any trial, and providing participants and potential participants with the appropriate level of information is important
Practical	Planning	Practical issues to consider when planning a trial/before conducting a trial with an adaptive design and health economic analysis	Who will need training and what sort of training would be appropriate? The level of complexity in the methods needs to be acceptable to funding panels reviewing grant applications and the agencies reviewing the evidence The role of public involvement Context where you could implement these methods from a practical point of view
	Implementation and conduct	Practical issues to consider when conducting or implementing a trial with an adaptive design and health economic analysis	How should interim decision-making be handled and what influence should competing factors have? Issues with cost data that might impact the practical application of this work Development of guidance documents for researchers implementing these methods
	Documenting and reporting	Practical issues to consider when reporting and writing documentation for a trial with an adaptive design and health economic analysis (not just reporting when trial has ended)	Documentation required throughout the trial process such as what to include in a protocol and analysis plans (pre-specification) Additional reporting requirements when publishing this research Reporting requirements for interim analyses

The framework was informed by existing literature [35, 44–57], discussions with the public advisory group and early data collected

We used a purposive sample to identify the views of a range of key stakeholders in the HTA process. Members of the public were recruited regardless of their experiences as a patient, a user of the National Health Service (NHS) or a participant in clinical trials. We also approached researchers and decision-makers involved in HTA, especially those who might use the proposed methods.

Members of the public were first contacted through a public involvement co-ordinator at a clinical research unit at a local hospital, a departmental public involvement lead and the public advisory panel supporting the research project. After the initial data collection, to extend the reach beyond members of the public with research experience, a message was posted to an online discussion forum (Sheffield Forum). Researchers and decision-makers were identified by contacting researchers known to be working in relevant areas such as prominent health economists, statisticians and clinicians.

Semi-structured interviews and focus groups were used to collect data. Data collection with members of the public was conducted face to face at the University of Sheffield where possible; telephone interviews were available to avoid excluding members of the public who were unable to travel. Interviews were the preferred approach for researchers and decision-makers, to reduce the burden on participants, as previous related research reported difficulties in engaging health economists [13].

Separate study documentation and topic guides were produced for the public and researcher groups reflecting the technical language and information provided to the respective participants. The researcher topic guide was piloted with a colleague. We implemented recommendations from the project’s public advisory group on how to modify the topic guide for members of the public.

Prior to data collection, all participants were sent a short video introducing the key concepts of adaptive designs and health economic analysis in plain English to allow full participation in the study [14]. This video was created with the support of the public advisory group so as not include any of LF’s preconceived ideas which might influence the subsequent responses of the participant.

All interviews and focus groups were conducted by LF and lasted between 30 to 60 mins and 60 to 90 mins respectively. The interviews and focus groups were audio recorded. The interviews were anonymised and transcribed. Demographic data were collected using an online Google Form.

Based on the literature [15–20], advice from the public advisory group, and keeping time constraints in mind, a target of a maximum of 20 researchers and 20 members of the public was set. Focus groups were planned to have a maximum of seven participants. At each stage the demographics of the sample were reviewed and subsequent participants selected to give a balanced and representative sample where possible, similar to the approach recommended by Francis et al. (2010) [21].

Transcriptions were imported into NVivo (QSR International v11, Melbourne, Australia). A framework analysis approach was adopted [22] and followed the recommended stages [23]:

1. Familiarisation with the data using the transcripts and any notes made during data collection
2. Coding the transcripts line by line and assigning a code from the a priori thematic framework or a new code
3. Updating the thematic framework with any new codes emerging from the data
4. Indexing transcripts using the categories and codes from the thematic framework
5. Charting data into a matrix summarising the data from each participant relevant to each of the categories from the thematic framework
6. Interpreting the results in the context of the research aims.

This included discussion of the findings with the public advisory panel. A table of recommendations was also sent to all study participants, who were given the opportunity to comment and suggest changes.

Ethics approval was granted by the University of Sheffield, School of Health and Related Research Ethics Committee (ref 009699). All participants were issued an information sheet and were required to sign a consent form or give verbal consent before data collection. The COREQ (Consolidated criteria for reporting qualitative research) checklist has been used in the reporting of this study [24] (see Additional file 1).

## Results

### Description of participants

In total, 29 participants took part in the study between October 2017 and March 2018. At this point it was felt that few new themes were emerging and a range of participants had been included. We conducted 18 one-to-one interviews and two focus groups. There were no repeat interviews. The participant characteristics are summarised in Table 2. One researcher did not complete the demographic survey. There were $\frac {2}{13}$ female researchers and $\frac {8}{15}$ female members of the public. Researchers predominantly came from statistical or health economic areas of expertise from both the private and public sectors. Two of the 13 researchers were based in the USA. Ten of 15 members of the public had participated in research through public involvement groups and $\frac {6}{15}$ had participated in a clinical trial.

**Table 2:** Demographics of participants who took part in interviews or focus groups. One researcher did not complete the demographics questionnaire

**Question**	**Response**	**Public** (*n*=15)	**Researcher** (*n*=13)
Gender	Female	8	2
	Male	7	11
Ethnicity (free text)	White, European	1	0
	White	3	3
	White British	8	7
	English	1	0
	Asian	1	1
	British	1	1
	Caucasian	0	1
Age (years)	30 or younger	1	1
	31–35	0	3
	36–40	5	2
	41–45	0	2
	46–50	1	2
	Older than 50	8	3
Highest academic qualification	Doctorate	1	8
	Post-graduate degree (Masters)	2	5
	Registered nurse & registered midwife	1	0
	Undergraduate degree	6	0
	Undergraduate degree, teaching certificate	1	0
	General Certificate of Secondary Education	1	0
	NA	3	0
Have you ever participated in a clinical trial?	No	9	-
	Yes	6	-
Have you ever been a member of a public involvement group?	No	5	-
	Yes	10	-
Experience in clinical trials research (years)	11–15 years	-	5
	16–20 years	-	1
	5–10 years	-	1
	Less than 5 years	-	2
	More than 20 years	-	3
	NA	-	1
Current employment sector	Private	-	6
	Public	-	7
Location	United Kingdom	-	11
	United States of America	-	2
Area of expertise (can choose multiple)	Clinician	-	1
	Health economics	-	8
	Health services research	-	1
	Value of information analysis	-	2
	Adaptive designs	-	3
	Clinical trial management	-	1
	Statistics	-	5

The health economists interviewed had limited experience with adaptive designs and reported that they are frequently not consulted in the design of clinical trials. Researcher participants suggested that health economists and statisticians frequently work independently, with their key point of contact often being the trial chief investigator. However, study participants did not feel this negatively impacted their work.

The knowledge and experience of members of the public who took part in the study came predominately from three main sources: the media, involvement in research as public advisors or participation in a clinical trial. Participants acknowledged that the media was likely to give biased views on healthcare decision-making for the general public and there was perhaps a lack of trustworthy resources available.

### Current practice

Increasing the prominence of cost-effectiveness; improving the quality of health economic analyses; and preventing adaptive designs stopping before there is sufficient evidence for an accurate health economic analysis were suggested by study participants as some of the advantages of using health economics in adaptive designs. A study participant suggested that while formal VOIA calculations are rare, informal ‘rules of thumb’ based on the same ideas are commonplace. Developing decision rules for cost-effectiveness of the research similar to the rule for clinical effectiveness (*p* <0.05) and cost-effectiveness of the intervention (willingness-to-pay thresholds) would help the practical interpretation and implementation of this approach.

Few participants had seen the use of health economics in the design and analysis of adaptive clinical trials in practice. It was felt that further work is needed to extend the existing theory to the real-world context, and concerns were raised that combining these two complex methods might be deemed too challenging and hinder their uptake. It was also suggested that improvements to the methods of VOIA were required to provide accepted standards such as the time horizon over which value of information is calculated. The use of adaptive designs and VOIA in trial design and research prioritisation would need to be more commonplace before adding further complexity and combining the two approaches in a trial.

…*putting both together is going to be doubly complicated and time consuming and costly and so on* …P35 Health economist

### Ethical considerations

Participants in the qualitative study agreed that clinical effectiveness is the main aim of a clinical trial, whether it is adaptive or a fixed sample size design. Both researcher and public participants could appreciate the importance of demonstrating cost-effectiveness in a trial; however, they felt it was still important to answer the clinical question first.

*It feels to me like it might be unfortunate to move into a world where, where clinical research, in general per se, was shaped too much, designed too much with economic considerations.*…P19 Member of the public

On the other hand, some participants argued that cost-effectiveness decisions provide unseen benefits in healthcare. For example, if a trial stopped early on cost-effectiveness grounds, this would allow resources to be spent elsewhere in a more cost-effective way, conferring benefits to participants outside of the clinical trial. One researcher suggested that incorporating health economics into the adaptive design changes the focus of the trial from the individual to a population level. However, others felt this might be deemed unacceptable to some stakeholders in the HTA process.

A number of the participants agreed that a health economist should be included on a data monitoring and ethics committee (DMEC) if health economics is used as part of the design and analysis of an adaptive trial. On the other hand, one researcher argued that funding decisions should be made by the funder, and it would not be appropriate to delegate this responsibility to the DMEC.

…*the DMEC have a very specific role looking at safety* […]. *But once you get into value of information that’s the funder’s decision and I don’t think the funder should delegate that to the DMEC.* P1 Funding panel member

### Methodological considerations

A number of participants were concerned about how the volatile nature of cost data would be handled if interim adaptations were made based on cost-effectiveness. This adds complexity when deciding whether to modify (or even stop) a trial on a given day when an interim analysis takes place, as this may be the wrong decision if, for example a drug price decreases.

*There’s still this question of whether it is cost economic now as per tomorrow, so you might miss an opportunity.* P6 Statistician

Study participants also highlighted that global multi-centre trials may encounter difficulties when using cost-effectiveness to inform interim adaptations. An interim analysis in one centre might demonstrate sufficient evidence to stop the trial early based on the cost-effectiveness rules in their jurisdiction, but this might not be reflected in the other centres around the world. Additionally, the extent to which adaptive trials incorporate health economics in the private sector might depend on whether the focus is on the US market or other countries where cost-effectiveness plays a more prominent role, such as the UK.

It was unappealing to many participants, that an adaptive design could stop early based on the cost-effectiveness of the intervention or the research itself. Alternative uses for the interim data suggested by participants included:

1. A hierarchy of stopping rules, where the clinical outcome is the first consideration in the adaptive decision-making and then, dependent on this result, cost-effectiveness may also be used to inform modifications to the trial.
2. The trial could be modified based on health economic grounds rather than the more extreme case of stopping a trial.

### Practical considerations

Study participants felt that successfully implementing this approach would require sufficient resourcing, careful planning and also building a study team of researchers and clinicians who have sufficient training and are supportive of the approach. Adding the health economic layer to adaptive clinical trials raised a resourcing problem for many participants, as more time and resources will be required before a project has been funded. In the private sector, this type of impact was thought to vary depending on the size of the company.

Funding panels and HTA committees are composed of non-experts who will require sufficient training in the methods to allow them to assess their appropriateness in answering the research question and to interpret and assess the quality of the evidence. Study participants highlighted the value of developing software to help make methodology accessible and increase its use. Case studies were suggested as a way of demonstrating the value of using health economics in adaptive clinical trials. This could increase the understanding of the methods and appease the concerns of many participants regarding the complexity of this approach. The methodology would need to be presented in an accessible way for non-experts.

…*the crucial thing is that the analysis has to be very understandable because* […] *in practice it will be unhelpful if a value of information analysis was updated and appeared to everyone involved in the trial to be a black box analysis.* P1 Funding panel member

It will be increasingly important for the health economic analyses to be pre-specified if used to inform adaptive decision-making during a trial. There would need to be a greater level of scrutiny of the health economic analyses. However, concerns were also raised by participants about the potential challenge of pre-specifying everything for a health economic analysis prior to any trial results. Participants working in industry were concerned about the impact of early examinations of the data on cost-effectiveness grounds and the requirements to publicly report all analyses.

…*it’s sort of …you’ve got chicken and egg here.* […] *Your model is developed as you look at the data but I think it would be quite difficult to do that because, yeah they sort of evolve alongside each other*P39 Health economist

## Discussion

Members of the public, researchers and decision-makers identified ethical, methodological and practical issues associated with the use of health economics in adaptive clinical trials. Recommendations and proposed actions were drawn from the themes identified in the data, reflecting the differing views of stakeholders in the HTA process and experiences of the research team and public advisory group. Recommendations are discussed under the three key themes—ethical, methodological and practical—and are summarised in Table ??.

**Table 3:** Summary of key recommendations and proposed actions from the interviews and focus groups with members of the public, researchers and decision-makers

**Issue**	**Recommendation**	**Action**
Clinical effectiveness is the main focus when thinking about the aims of a clinical trial	The importance of clinical effectiveness should be reflected in the development of methods for using health economics in adaptive trials. Possible approaches include: •Using early examinations of the trial to check that all health economic data are being collected as required •Using early trial data to update the health economic model •Using a hierarchy of interim decision rules where any decisions made based on cost-effectiveness depend on decisions made about clinical outcomes •Only considering health economic outcomes at later examinations of the data •Using health economic data to make modifications to the trial such as increasing the sample size but not major changes such as stopping the trial early	Explore how existing methods for the use of health economic-based stopping rules would work in the real-world setting, by applying the methods to a diverse range of case studies
Study participants appreciate the importance of cost-effectiveness to decision-makers, but they consider this to be secondary to clinical effectiveness	There needs to be a change in the mentality of the research community towards the role of health economics and cost-effectiveness in healthcare decision-making	Development of materials to educate on the importance and ethical motivations for thinking about cost-effectiveness. This could be written with members of the public and developed into workshops, online materials and leaflets
Stakeholders may not be familiar with the methods of adaptive designs or value of information analysis and their potential advantages and limitations	Develop software and tutorial style case studies for researchers to help them understand the methods and allow them to interpret the results of trials using this approach or use these methods in their own research Development of plain English summaries and case studies highlighting the impact of the methods on patients and the public	A Practical Adaptive and Novel Designs and Analysis (PANDA) Toolkit is under development that aims to provide researchers with training materials on adaptive design clinical trials [58]. This could include materials aimed at members of the public Organise workshops (such as at conferences) for researchers, highlighting the potential for the methods to be used together and issues to consider
If health economics is to inform decisions made using early examinations of data, there may be a need for this specialist knowledge on trial committees	Include health economists on DMECs where health economics is used as part of the design and analysis of adaptive trials	Existing resources that help research teams identify DMEC statisticians, such as StatLink [40], could be extended to identify health economists. All DMEC members could be paid for their contribution and time Use mock DMECs to allow members to review the health economic and clinical data and see where issues with using the health economic data arise
Using health economics in the design and analysis of adaptive trials will require more work before the trial is funded, when researchers may not be paid for this work	Funding bodies should provide alternative funding options that allow researchers to develop new designs Researchers could include time at the start of a study to fully develop an adaptive trial design that uses health economics Researchers should look for methodology grants to fund the development of designs	Groups representing statisticians and health economists (such as the MRC Adaptive Designs Working Group and ISPOR—the Professional Society for Health Economics and Outcomes Research) should work together to persuade funders and regulators of the need for alternative ways to fund adaptive clinical trials and the benefits this will have to health research and maximising limited research budgets
There is currently limited interaction between health economists and statisticians working on trials and more generally between the two research communities	Encourage statisticians and health economists to work together and increase communication to facilitate the implementation of health economics in adaptive trials by sharing expertise	Locally, health economists and statisticians working on the same clinical trial should have regular meetings throughout the study. Nationally, there could be joint events between groups such as the NIHR Statistics Group and the Health Economic Study Group to discuss common issues and encourage training in statistics for health economists and health economics for statisticians
If health economics is to be used in adaptive clinical trials, the methods need to be outlined in advance so the results of the trial are still valid and robust	Before the trial begins, researchers should outline how health economics is going to be used in the trial and how the early examinations of the data will be used to calculate cost-effectiveness and inform decision-making	Extend current work developing guidance for health economic analysis plans to think about specific issues that might arise in adaptive clinical trials [42]. This will likely require health economists to have some experience of working on an adaptive trial
Calculating the costs of conducting an adaptive clinical trial can be complicated. For example, one must provide justifications of costs and cost projections in a grant application	It is important to understand the costs of conducting an adaptive trial such as the costs of finding patients, training staff and analysing data so that one can compare adaptive and non-adaptive trial designs and inform stopping rules based on health economics	Develop a standardised approach for calculating the costs of an adaptive clinical trial, illustrated using a case study

### Ethical considerations

Currently the use of health economics in the design and analysis of adaptive and non-adaptive clinical trials is largely secondary to clinical outcomes [11]. Study participants felt that for health economics to play a greater role there needs to be a shift of emphasis from the ethical perspective of beneficence—the duty to allocate resources to benefit the individual [25]—to the broader perspective of distributive justice: allocating resources to benefit the whole population [26, 27]. Changing the status quo requires the public and policy makers to find compelling ethical arguments showing that cost-effectiveness decisions provide an unseen benefit [28]. We recommend developing unbiased and simple materials on adaptive designs and health economics. Public advisors could be used to develop materials and consent forms that explain the methods of adaptive designs and health economics in sufficient detail for potential trial participants to make an informed decision to participate, as well as highlighting the broader implications of taking part in the research.

The acceptability of the role of cost-effectiveness to stakeholders is crucial when considering the direction of methods development. It is fruitless to develop statistical methods that incorporate health economics into an adaptive design if this is never implemented in practice due to concerns over the role of cost-effectiveness. For example, patients may be unwilling to be randomised, as they do not agree with decision-making centred on cost-effectiveness. Based on discussions with the study participants and the public advisory group, we recommend exploring alternative approaches, including the following:

- Interim analyses used to check that all health economic data are being collected as required
- Interim data used to update the health economic model
- A hierarchy of interim decision rules where the health economic decisions are dependent on the outcome of the clinical effectiveness analysis
- Health economics decision rules that are only considered at later interim analyses, giving the advantage of more mature data and also more weight to clinical outcomes at the start of the trial
- Health economics used to make less extreme modifications such as increasing the sample size based on an expected value of sample information calculation.

The choice of approach could be made in collaboration with a public advisor so that the identified approach is acceptable to patients.

### Methodological considerations

The advantages of using health economics in adaptive designs include saving resources and preventing patients from being needlessly randomised [11, 29, 30]. Such an approach will formalise the informal rules of thumb described by a study participant as commonly used by decision-makers and funding panels, so that they can be applied in an objective and consistent manner. We suggest that developing decision rules for cost-effectiveness of the research, similar to the rule for clinical effectiveness (*p* values) and cost-effectiveness of the intervention (willingness-to-pay thresholds), would help the practical interpretation and implementation of this approach. Public advisors could be used to ensure these rules are appropriate and reflect the views of patients and the public.

It will also be important to consider the limitations of both methodologies, including the potential for adaptive designs to introduce bias in the estimation of point estimates and confidence intervals and maintaining appropriate levels of blinding during interim analyses so as not to influence the conduct of the trial [31, 32].

The methods of adaptive designs and value of information analysis (VOIA) are not commonplace in HTA [10, 33, 34]. It will be vital to address the limitations of these methods to successfully implement them together in clinical trials. As identified by the study participants, these methods could be seen as more complex, potentially acting as a barrier to their use in practice. Work in the field of adaptive designs to promote their use and understanding amongst the health research community includes:

- The Adaptive Designs Working Group, part of the Medical Research Council (MRC) Hubs for Trials Methodology Research, which aims to increase implementation of adaptive designs through tutorial papers in mainstream medical journals (see, e.g. [32]), development of software and presentations and lectures
- The Adaptive CONSORT Extension (ACE) project, which aims to enhance transparency, credibility, reproducibility and replicability of adaptive trials by developing a consensus-driven extension to the CONSORT (Consolidated Standards of Reporting Trials) statement specific to adaptive designs [35].

Measures for increasing the use of value of information analyses include:

- The Professional Society for Health Economics and Outcomes Research (ISPOR) Value of Information Analysis for Research Decisions task force, which aims to develop good practice guidance for using methods of value of information analysis to inform both technology reimbursement decisions and research prioritisation decisions [36]
- Collaborative Network for Value of Information (ConVOI) group, an international network working to improve the calculation, adoption and application of value of information methods in clinical and public health research [37, 38].

We recommend the use of similar initiatives to promote the use of health economics in the design and analysis of adaptive clinical trials.

### Practical considerations

If the use of health economics becomes common in the analysis of adaptive designs, then guidance on the composition of DMECs [39] should be updated, and lists which help identify DMEC members—such as StatLink [40]—could be extended to include health economists.

Such an approach will have cost consequences for clinical trials with the involvement of health economists throughout. There will be a requirement to conduct more analyses such as model conceptualisation prior to the trial being funded [13]. However, the development of a health economic model and cost-effectiveness analyses based on systematically reviewed current evidence is an essential step prior to undertaking any trial regardless of its design [41]. In the public sector, where there are few resources to bridge grant funding, researchers would need to include the costs of trial design within grant applications. Groups representing statisticians and health economists will need to persuade funders and regulators of alternative ways to fund adaptive clinical trials and value of information analyses.

If health economics is to be successfully implemented as part of an adaptive clinical trial, we recommend that trial sponsors need to ensure integrated working between health economists and statisticians throughout the trial process. More generally, across the research community links between statisticians and health economists could be encouraged through networking events and joint workshops to identify common ground and explore ways of working together more efficiently.

Pre-specification of health economic analyses will be crucial in maintaining the validity and integrity of adaptive designs that use health economics, with analysis plans including a description of the monitoring and adaptation plan, as well as pre-specification of methods used at interim analyses [29, 42]. The timing of interim analyses must be realistic given their complexity, and their blinding must be carefully considered. Cross-disciplinary training materials will be required, supplemented by case studies on the use of health economics in sequential trials [43], to raise awareness and advance methodological development by highlighting practical problems with their application.

### Strengths and limitations

This is the first qualitative study to explore the ethical, methodological and practical issues of using health economics in the design and analysis of adaptive clinical trials. Participants came from different backgrounds and with experiences across the healthcare decision-making process. This study included a number of health economists, a group previously found to be hard to reach in adaptive designs research [13]. Members of the public were an important stakeholder group in this research, as the ultimate beneficiaries of clinical trials and healthcare decision-making that uses an adaptive design and health economic analysis. Additionally, members of the public are all potential participants in trials that use these methods. Failing to reflect their views in the design and analysis of clinical trials could, for example, make it difficult to recruit to trials that use an unpopular design based on cost-effectiveness.

Few female researchers took part in the study; however, it is not felt that the gender of the participants is likely to influence their responses. Instead, participants’ knowledge and experiences are more likely to inform their comments. Further work could consider a larger sample size and a broader range of experience of participants, such as programme managers at funders such as the National Institute for Health Research.

A public advisory group played an intrinsic part in the design and analysis of the qualitative study. The group were given training in adaptive designs and health economics by LF. They provided feedback on the information sheet, consent form and topic guide for members of the public to ensure the questions and proposed plan were suitable. The group developed the script for the short video sent to all study participants. After analysis was complete, the group met again to discuss the results and check the interpretation of findings. Where appropriate, their interpretation has been embedded in the paper.

A limitation of the research is that the members of the public and non-expert researchers and decision-makers were given a top-level understanding of the topics of value of information; thus, their responses are considering a broad view of cost-effectiveness considerations in clinical trials. However, the views of participants are still important in understanding the priorities of the stakeholder groups around the role of cost-effectiveness in adaptive clinical trials. Further work could consider a more detailed explanation of the methods of value of information analysis, providing scenarios of potential roles for the methods in adaptive clinical trials and using these to explore the views of stakeholders with a deeper understanding of the method.

### Conclusion

The use of health economics in the design and analysis of adaptive clinical trials has the potential to increase the efficiency of health technology assessments worldwide. However, careful consideration is needed to ensure that the statistical methods reflect the importance of clinical effectiveness to stakeholders and that adequate training and resources are provided to facilitate the implementation of this approach.

## Supplementary information

**Additional file 1** Completed COREQ checklist.

## Data Availability

The data from the current study are available from the corresponding author on reasonable request.

## Abbreviations

ACE: Adaptive CONSORT Extension
CONSORT: Consolidated Standards of Reporting Trials
COREQ: Consolidated criteria for reporting qualitative research
DMEC: Data monitoring and ethics committee
HTA: Health technology assessment
ISPOR: The Professional Society for Health Economics and Outcomes Research
MRC: Medical Research Council
NHS: National Health Service
PANDA: Practical Adaptive and Novel Designs and Analysis (Toolkit)
QALY: Quality-adjusted life year
VOIA: Value of information analysis
